# Clinical and genetic features of syndromic craniosynostosis in 18 Chinese probands: novel candidate genes and phenotypes of known pathogenic genes

**DOI:** 10.3389/fgene.2026.1876428

**Published:** 2026-08-04

**Authors:** Yufeng Huang, Lingyue Huang, Lilin Hu, Li Tan, Peiwei Zhao, Sukun Luo, Ting Yu, Yanqiu Hu, Yuanzhi He, Hao Du, Xuelian He

**Affiliations:** 1 Genetics and Precision Medical Center, Wuhan Children’s Hospital (Wuhan Maternal and Child Healthcare Hospital), Tongji Medical College, Huazhong University of Science and Technology, Wuhan, China; 2 Clinical Medical Research Center for Birth Defect Prevention and Treatment in Wuhan, Wuhan, China; 3 Department of Neurosurgery, Wuhan Children’s Hospital (Wuhan Maternal and Child Healthcare Hospital), Tongji Medical College, Huazhong University of Science and Technology, Wuhan, China; 4 Department of Pediatric Emergency, Wuhan Children’s Hospital (Wuhan Maternal and Child Healthcare Hospital), Tongji Medical College, Huazhong University of Science and Technology, Wuhan, China

**Keywords:** idiosyncratically, NOG, Pqbp1, pten, SRCAP, whole-exhume sequencing

## Abstract

**Introduction:**

Craniosynostosis is a common congenital disorder characterized by premature fusion of one or more cranial sutures, categorized into non-syndromic (NSCS) and syndromic craniosynostosis (SCS). SCS accounts for ∼30% of cases, often accompanied by severe clinical complications, with 20%–25% of patients lacking a clear molecular etiology.

**Methods:**

We enrolled 18 Chinese SCS patients and analyzed their clinical and genetic data via whole-exome sequencing (WES), copy number variation (CNV) analysis, and Sanger sequencing validation.

**Results:**

We identified 15 single nucleotide variants, 2 insertions/deletions, and one microdeletion at 11q23.3q25. Thirteen probands harbored pathogenic/likely pathogenic variants in known craniosynostosis-related genes: *FGFR2* (n = 4), *FGFR3* (n = 2), *TWIST1* (n = 2), and one each in *ERF, EFNB1, SON, HUWE1*, and *KRAS*. Three probands carried variants in *PTEN, SRCAP, PQBP1*, none of which have previously been associated with craniosynostosis. Notably, a *de novo* nonsense variant (c.562G>T, p. Glu188Ter) was identified in NOG, a promising candidate gene for craniosynostosis.

**Discussion:**

Our findings broaden the genotypic spectrum of craniosynostosis and further highlight the genetic heterogeneity underlying this disorder. The discovery of variants in *PTEN, SRCAP, PQBP1*, and *NOG* identifies novel candidate genes and uncovers potential disease mechanisms.

## Introduction

Premature ossification of one or more cranial sutures (craniosynostosis) is a common craniofacial anomaly (1 in 2000–2500 live births), classified into syndromic (SCS, ∼30%) and non-syndromic (NSCS, >70%) subtypes based on additional extracranial abnormalities. SCS can cause severe neurological sequelae by restricting brain growth and elevating intracranial pressure (Wu and Gu, 2019). Hence, identification of causes and underlying mechanisms would aid the understanding of these conditions.

Aberrant suture fusion is driven by dysregulated signaling pathways (FGF/FGFR, TGF-β/BMP, etc.) and mutations in genes such as *FGFR1-3*, *TWIST1*, and *RUNX2* (Wu and Gu, 2019; Yapijakis et al., 2023), with recent implications of chromatin modification and retinoic acid signaling genes (Timberlake et al., 2023). Over the last decades, whole-exome sequencing (WES) has identified over 50 causal/relevant genes, and OMIM (http://www.omim.org) documented 205 craniosynostosis-related syndromes by 17 April 2026. However, 20%–25% of SCS cases lack identified causative variants, highlighting genetic knowledge gaps (Kutkowska-Kazmierczak et al., 2018).

To address this gap, we retrospectively analyzed 18 SCS patients (Wuhan Children’s Hospital, 2018–2025) who underwent trio WES/copy number variation sequencing (CNVseq). We first report a *de novo NOG* variant (novel candidate gene) and three probands with *PTEN*, *SRCAP*, or *PQBP1* variants, representing unreported phenotypic expansions (Nowaczyk et al., 1993). These findings expand craniosynostosis’ genotypic spectrum and underscore its genetic heterogeneity.

## Methods

### Study cohort

**FIGURE 1:**
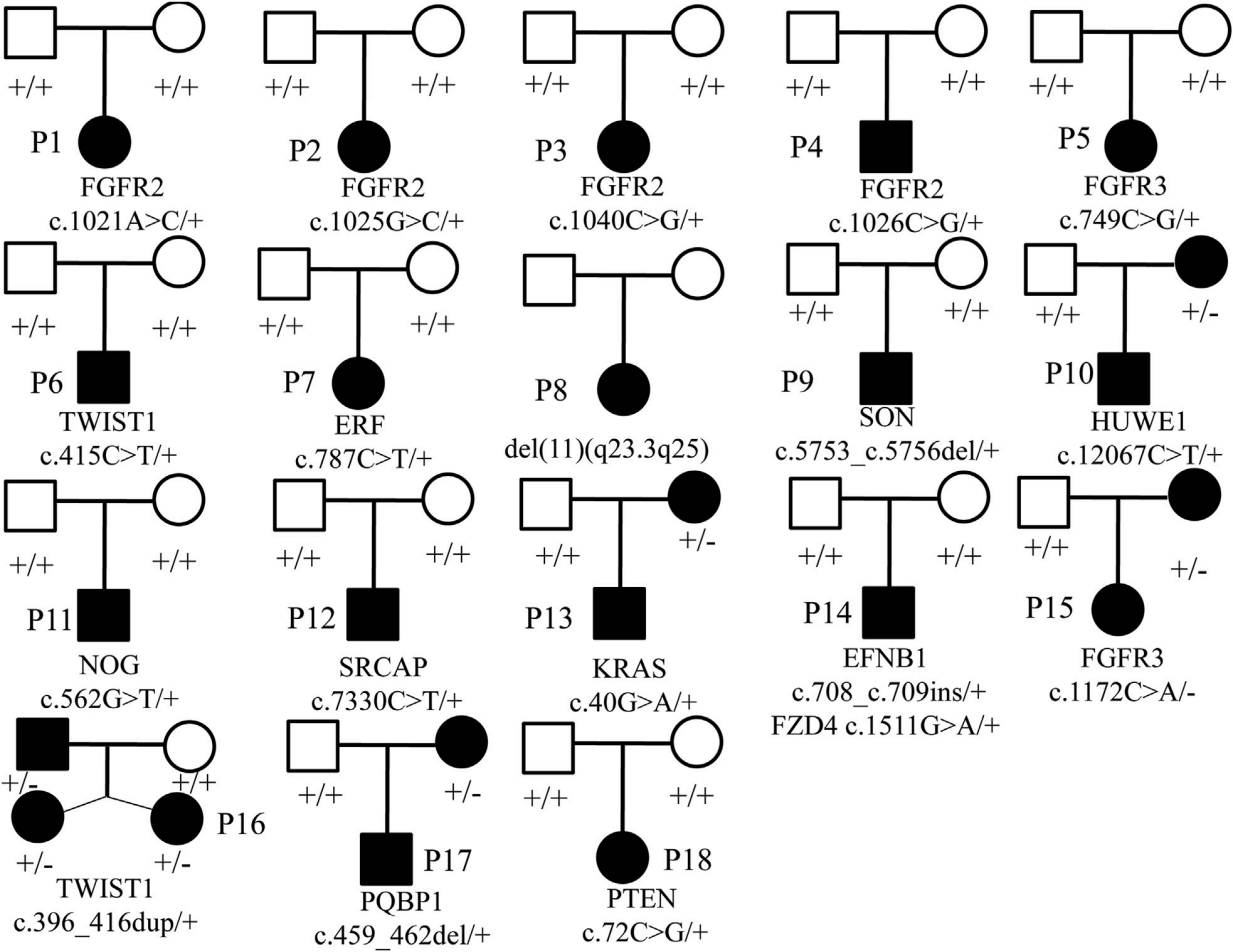
Pedigrees of 18 families and the genetic causes identified in the patients.

We retrospectively analyzed a total of 5349 patients suspected of genetic disorders who underwent WES/CNVseq in Wuhan Children’s Hospital from August 2018 to October 2025, identifying 18 SCS probands with causative genetic variants or chromosomal abnormalities (Figure 1). Craniosynostosis was confirmed by physical examination and/or three-dimensional computed tomography (3D-CT). This study was approved by the Ethics Committee of Wuhan Children’s Hospital (Approval NO: 2020R006-E05). Written informed consent was obtained from all participants’ legal guardians; consent was waived for deceased probands per IRB protocols.

### WES and variant validation

Genomic DNA was extracted from leukocytes using the QIAamp DNA Blood Maxi Kit (Qiagen, Germany). WES and subsequent variant analysis were performed at Chigene (Beijing) Translational Medical Research Center, following protocols similar to our previous studies (Tan et al., 2021; Huang et al., 2024). Bioinformatics analysis involved raw read trimming (Trimmomatic v0.39), alignment to GRCh37/hg19 (BWA-MEM v0.7.17), duplicate marking (Picard v2.19.0), base recalibration, and germline variant calling (GATK HaplotypeCaller v4.5). Variants were annotated (ANNOVAR v2021-05–20), filtered against population databases (gnomAD v3.1.2, 1000 Genomes Phase three), and assessed for functional impact using ten prediction algorithms (SIFT, PolyPhen-2, PROVEAN, CADD v1.6, etc.), then classified per American College of Medical Genetics and Genomics/Association of Molecular Pathology (ACMG/AMP) guidelines or ClinGen PTEN Variant Curation Expert Panel (VCEP) specifications (Kalia et al., 2017) into five categories: Pathogenic, Likely Pathogenic, Variant of Uncertain Significance, Likely Benign, and Benign. All candidate variants were confirmed via bidirectional Sanger sequencing (ABI 3730xl, Thermo Fisher). The variants were described according to current Human Genome Variation Society (HGVS) recommendations and report variants using the Matched Annotation from NCBI and EMBL-EBI (MANE) Select transcripts. Patient clinical phenotypes were annotated using standardized HPO terms, which were implemented for phenotype-driven variant prioritization and filtering throughout the WES analysis workflow.

### CNVseq

The CNVseq methodology employed in this study is similar to WES but utilizes a low-coverage whole-genome sequencing (WGS) strategy to achieve comprehensive genomic coverage. This approach enables the detection of large-scale CNVs across the genome. The detailed protocol was as previously described (Luo et al., 2021).

### Functional enrichment analysis

Protein-protein interaction (PPI) network analysis using the STRING database (https://string-db.org/), a comprehensive resource that integrates experimentally validated interactions, computational predictions, literature mining, and co-expression data across thousands of species. The Metascape database (https://metascape.org) was used to perform GO functional enrichment analysis for the key module genes (Zhou et al., 2019). The criteria of the analyses were p-values <0.01, min (overlap) = 3, and min (enrichment) = 1.5, and the outcomes were visualized using enrichment network, sankey diagram and dot bubble.

## Results

### Clinical description

A total of 18 unrelated probands with SCS were included. Detailed clinical information, including the clinical types and manifestations of craniosynostosis, family history, brain CT/MRI, and other relevant examinations, were summarized in Table 1.

**TABLE 1:** Clinical features of the 18 craniosynostosis probands.

No.	Gender	Age	Suture(s)	Craniofacial futures	Neurological symptoms	Ocular abnormalities	Cardiovascular defects	Genitourinary anomalies	Others	CT	MRI	Surgical treatment
P1	F	1Y2M	Metopic	Abnormal head shape, ocular proptosis, orbital hypertelorism, mild flattened nose, blepharotosis, hydrocephalus	Developmental delay	No	No	No	Lacrimal duct eobstruction, congenital choanael stenosis	Proximal occlusion of the four ventricles	Occipital venous anomaly, enlarged third and bilateral lateral ventricles	Yes, 2Y
P2	F	10D	Sagittal	Microcephaly, frontal bossing, ocular proptosis	Too young to assess	No	Patent ductus arteriosus, atrial septal defect (oval hole)	No	Neonatal necrotizing enterocolitis, neonatal gastric bleeding	Partially fused sagittal suture narrow cranial cavity	Slightly larger triangular area of bilateral lateral ventricle	Yes, 2 M
P3	F	1Y	Coronal, sagittal, rhomboid	Abnormal head shape, frontal bossing, low-set ears, open-mouth posture, sialorhea, entropion complicated with trichiasis	Developmental delay, syringomyelia	No	No	No	No	Frontal apical cranial protrusion; coronal, sagittal and rhomboid suture closure	Frontoparietal white matter signal abnormality, bilateral ventriculomegaly, septum pellucidum agenesis, C5–C7 vertebral signal abnormality, syringomyelia	Yes, 4Y
P4	M	10M28D	Bilateral coronal	Sharp head, ocular proptosis	No	Exotropia	No	No	No	Cerebellar tonsil descends; unclear the coronal suture and the bilateral squamosal sutures	Chiari deformity, abnormal signals in bilateral frontal white matter, slightly larger bilateral lateral ventricles	Yes, 1Y
P5	F	3Y7M	Coronal	Craniofacial asymmetry (asymmetric frontal, unequal palpebral fissures, left eye hypotelorism)	Developmental delay, speech delay, cognitive impairment	Strabismus	Central atrial septal defect (oval hole type)	No	No	Fused right coronal suture, smaller right frontal cranial cavity than the left	Abnormal signal shadow in bilateralfrontal parietal lobe, smaller right frontal cranial cavity	No
P6	M	11M16D	Coronal	Abnormal head shape, microcephaly, blepharoptosis, congenital narrow posterior nostrils, and low-set ears	Developmental delay	No	No	No	No	Fused coronal suture, wide anterior fontanelle	NA	Yes, 1Y
P7	F	1M17D	Sagittal	Abnormal head shape, hydrocephalus	Developmental delay	No	No	Unilateral kidney	No	Cerebellar subtonsillar hernia, fused sagittal suture	Slightly widened bilateral frontotemporal extracerebral space, slightly enlarged bilateral lateral ventricles, abnormal head shape	Yes, NA
P8	F	5D	NA	Dysmorphic features, auricular malformation, multiple swollen, deformed and stiff joints	Developmental delay	No	No	Malformation of external genitalia	No	NA	Abnormal brain morphology and multiple abnormal intracranial signal foci	Yes, 2Y
P9	M	1Y1M	Coronal	Right frontal flattening, right orbit retraction, left head tilt	Developmental delay, motor developmental delay, cognitive impairment, epilepsy	Strabismus	No	Cryptorchidism	Flat and outward-turned feet	Partially fused right coronal suture, smaller right cranial cavity than the left	Larger cisterna magna, bilateral lateral ventriculomegaly, widened right posterior fossa and bilateral temporal extracranial spaces, hypoplastic right anterior cranial fossa	Yes, 2Y
P10	M	4 M	NA	Microcephaly, premature fontanelle closure, prominent ears, micronathia, brachyury	Developmental delay (fetal growth restriction and DD), epilepsy	No	No	No	Short stature, feeding difficulty	NA	Slightly larger cisterna magna	No
P11	M	3 M	Frontal, sagittal, right coronal	Scaphocephaly	No	No	No	Hypospadias, undescended right testicle	Radial deviation deformity of the little finger, hypertonia	Abnormal skull morphology, long anterior posterior diameter, inward depression of bilateral cranial bones, fused frontal, sagittal and right coronal sutures	NA	Yes, 4 M
P12	M	1Y3M	NA	Abnormal head shape, dysmorphic features	Developmental delay	No	Pulmonary valve stenosis, patent ductus arteriosus, interventricular septal and right ventricular wall hypertrophy	NO	Hypomyotonia, dysphagia, malnutrition, thin subcutaneous fat	NA	NA	No
P13	M	6Y2M	NA	Dysmorphic features (long face, high-arched eyebrows, flat nasal bridge, ocular hypertelorism, anteverted nares, long philtrum, thin upper lip, low-set ears, posteriorly rotated ears), prominent eyes, ptosis, scaphocephaly	Developmental delay, intellectual disability, speech delay	Strabismus, doptic atrophy	No	Cryptorchidism	Hypomyotonia	NA	NA	NA
P14	M	6Y10 M	Coronal	Dysmorphic features, low and flat nose bridge	No	Astigmatism, hyperopia, familial exudative vitreoretinopathy	No	No	Hypertrichiasis, streblomicrodactyly	NA	NA	Yes, 3Y
P15	F	6 M	NA	Dysmorphic features, congenital narrow posterior nostrils, hydrocephalus	Delayed motor development	No	Ventricular septal defect, atrial septal defect, patent ductus arteriosus	No	Short stature, feeding difficulty, recurrent pneumonia	NA	NA	Yes, 3Y
P16	F	5Y9M	Coronal, sagittal	Skull malformation, brachycephaly, mildly prominent forehead, mild bilateral ocular protrusion	No	No	No	No	Short stature, scoliosis, flat and bulky right big toe	Abnormal skull morphology, craniosynostosis, discontinuity of the frontal bone and bone adjacent to the left jugular bulb	Abnormal skull morphology, reduced fronto-occipital diameter	No
P17	M	1Y6M	Coronal, sphenoparietal, sphenofrontal, squamous, occipitomastoid	Microcephaly, flat forehead, ocular ypertelorism, mild external ear dysmorphism	Developmental delay, cognitive impairment, delayed motor development	Astigmatism	Ventricular septal defect, atrial septal defect, patent ductus arteriosus, pulmonary hypertension	No	No	Craniosynostosis	Slightly widened extra-axial spaces in the bilateral frontotemporal regions, with a small amount of subdural effusion in the right frontotemporal region	Yes, 1Y
P18	F	2Y7M	Sagittal	Scaphocephaly with large head circumference	Developmental delay, delayed motor development, speech delay, autism spectrum disorder	No	No	No	No	Craniosynostosis	Enlarged perivascular spaces	No

No., number; F, female; M, male; NA, not available; Y, years; M, months; D, days.

**FIGURE 2:**
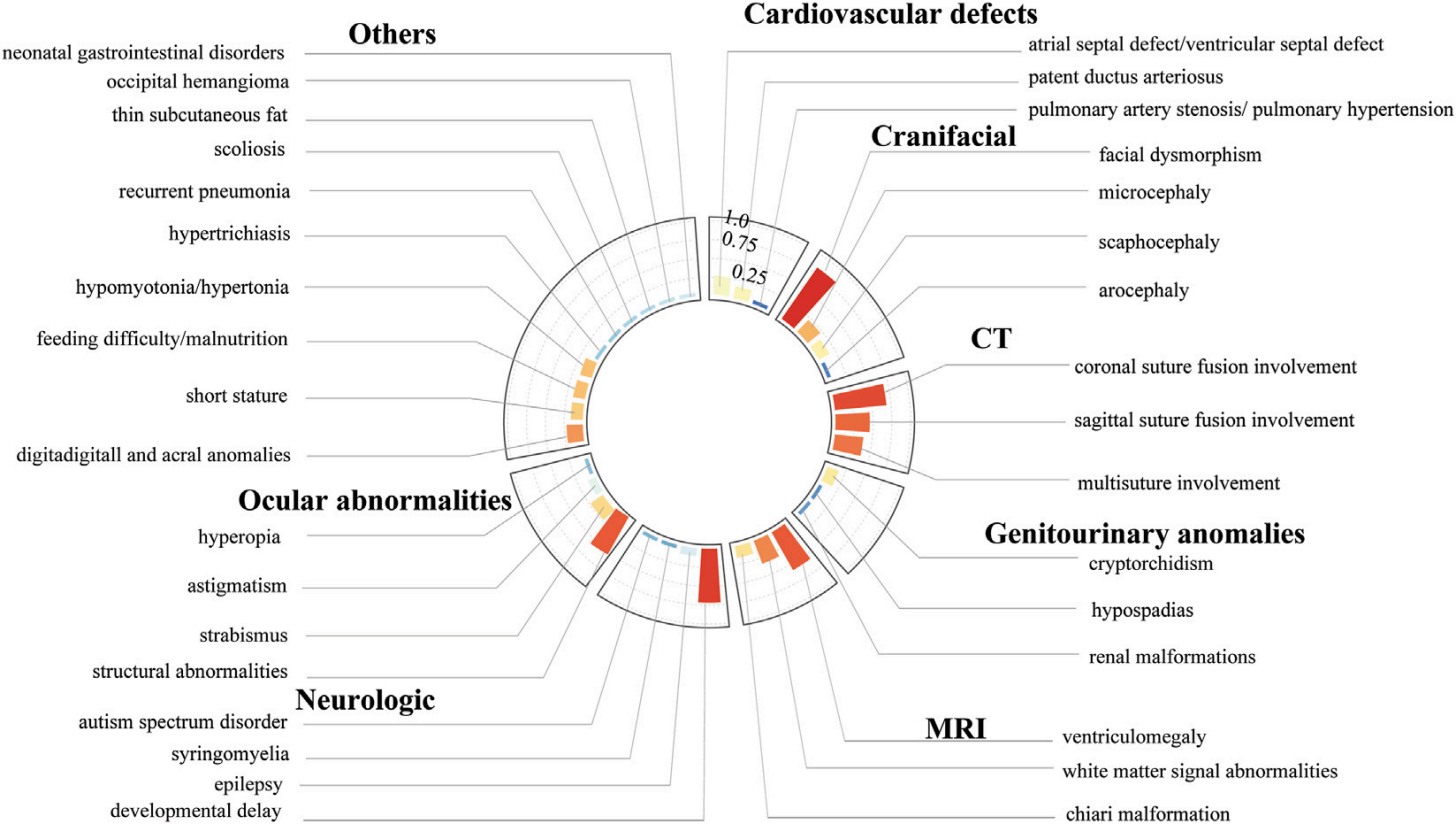
Circular bar plot representing frequencies for phenotypic features in the present cohort; bars are proportional to frequency and 25% intervals indicated by ticks.

Demographic analysis revealed equal gender distribution, with males and females accounting for 50% each, and 12 patients (66.67%) had undergone surgical intervention, with ages at surgery ranging from 2 months to 4 years. As presented in Table 2; Figure 2, among 13 patients with documented suture involvement, coronal suture fusion was most frequent (9/13, 69.23%), followed by sagittal suture fusion (6/13, 46.15%). Multisuture synostosis was observed in 38.46% (5/13) of patients; three sutures were fused in P3, P11 and P17, all involving the coronal suture fused. Five patients had not undergone surgery at the time of analysis, and relevant data were unavailable for one patient.

**TABLE 2:** Main phenotypic features of the present cohort.

Group	Phenotype	Frequency
CT	Coronal suture fusion involvement	9/13 (69.23%)
	Sagittal suture fusion involvement	6/13 (46.15%)
	Multisuture involvement	5/13 (38.46%)
MRI	Ventriculomegaly	7/12 (58.33%)
	White matter signal abnormalities	6/18 (33.33%)
	Chiari malformation	3/18 (16.67%)
Cranifacial	Facial dysmorphism	15/18 (83.33%)
	Microcephaly	4/18 (22.22%)
	Scaphocephaly	3/18 (16.67%)
	Arocephaly	1/18 (5.56%)
Neurologic	DD	13/18 (72.22%)
	Epilepsy	2/18 (11.11%)
	Syringomyelia	1/18 (5.56%)
	Autism spectrum disorder	1/18 (5.56%)
Ocular abnormalities	Structural abnormalities	10/18 (55.56%)
	Strabismus	4/18 (22.22%)
	Astigmatism	2/18 (11.11%)
	Hyperopia	1/18 (5.56%)
Cardiovascular defects	Atrial septal defect/ventricular septal defec	5/18 (27.78%)
	Patent ductus arteriosus	3/18 (16.67%)
	Pulmonary artery stenosis/pulmonary hypertension	2/18 (11.11%)
Genitourinary anomalies	Cryptorchidism	3/18 (16.67%)
	Hypospadias	1/18 (5.56%)
	Renal malformations	1/18 (5.56%)
Others	Digital/acral anomalies	4/18 (22.22%)
	Short stature	3/18 (16.67%)
	Feeding difficulty/malnutrition	3/18 (16.67%)
	Hypomyotonia/hypertonia	3/18 (16.67%)
	Hypertrichiasis	1/18 (5.56%)
	Recurrent pneumonia	1/18 (5.56%)
	Scoliosis	1/18 (5.56%)
	Thin subcutaneous fat	1/18 (5.56%)
	Occipital hemangioma	1/18 (5.56%)
	Neonatal gastrointestinal disorders	1/18 (5.56%)

Neuroimaging revealed consistent structural abnormalities (Table 2; Figure 2). Craniosynostosis was confirmed by CT in all 12 patients. Abnormal MRI findings were present in all these 12 patients, including ventriculomegaly (7/12, 58.33%), white matter signal abnormalities (4/12, 33.33%), and Chiari malformation (2/12, 16.67%). These imaging futures were closely correlated with neurological manifestations.

Craniofacial dysmorphism was universal (18/18, 100.0%), including facial dysmorphism (15/18, 83.33%), microcephaly (4/18, 22.22%), scaphocephaly (3/18, 16.67%), and arocephaly (1/18, 5.56%). Neurodevelopmental abnormalities were prevalent (13/18, 72.22%), DD (13/18, 72.22%), with additional cases of epilepsy (2/18, 11.11%), syringomyelia (1/18, 5.56%), and autism spectrum disorder (1/18, 5.56%). Ocular structural and functional abnormalities (11/18, 61.11%) included structural abnormalities (10/18, 55.56%), strabismus (4/18, 22.22%), astigmatism (2/18, 11.11%), and hyperopia (1/18, 5.56%). Cardiovascular defects occurred in five patients (27.78%), including atrial septal defect/ventricular septal defect (4/5, 80.0%), patent ductus arteriosus (3/5, 60.0%), and pulmonary artery stenosis/pulmonary hypertension (2/5, 40.0%). Genitourinary anomalies were detected in five patients (27.78%), including cryptorchidism (3/5, 60.0%), hypospadias (1/5, 20.0%), and renal malformations (1/5, 20.0%) (Table 2; Figure 2). Additionally, digital/acral anomalies (4/18, 22.22%), short stature (3/18, 16.67%), and abnormal muscle tone (3/18, 16.67%) were observed.

All 11 patients with ocular abnormalities were found to have concurrent craniofacial dysmorphism (e.g., proptosis, hypertelorism, and ptosis in P1). Cardiovascular defects co-occurred with neuroDD in 80% (4/5) of cases (e.g., DD, speech delay, and cognitive impairment in patient P5). Patient P11, who carried the *NOG* variant, exhibited hypospadias and fifth finger clinodactyly. Patient P8 manifested severe multisystem involvement, including joint contractures and external genitalia malformation (Table 2; Figure 2).

### Genetic findings

A total of 17 distinct pathogenic/likely pathogenic variants and one pathogenic CNV were identified as positive genetic diagnoses (Table 3; Figure 1). Most of these diagnoses (n = 10) involved variants in well-established SCS-related genes and signaling pathways: 4 *de novo* missense variants in *FGFR2*, 2 *FGFR3* variants (1 maternally inherited), 2 *TWIST1* variants (1 paternally inherited and 1 maternally inherited), 1 *EFNB1* variant, and 1 ERF variant. Patient P14, who carried the *EFNB1* variant, also presented with exudative vitreoretinopathy, which was explained by a *de novo FZD4* variant.

**TABLE 3:** Genetic causes detected in individuals with craniosynostosis in this study.

NO.	Gene, transcript and variant/CNV	ACMG class/Criteria	De novo	Have been reported/References
P1	FGFR2, NM_000141.5, c.1021 A>C, p.Thr341Pro	P: PS2+PS3-supporting + PS4-moderate + PM2-supporting + PP3-moderate	Yes	Yes, PMIDs: 7,719,345, 9586546, 16418739, 27028366
P2	FGFR2, NM_000141.5, c.1025G>C, p.Cys342Ser	P: PS2+PS3-moderate + PS4-moderate + PM1+PM2-supporting + PM5-strong + PP2+PP3-strong	Yes	Yes, PMIDs: 7987400, 8644708, 9586546, 10541159, 12884424, 12884434, 24127277, 25271085, 26362256
P3	FGFR2, NM_000141.5, c.1040C>G, p.Ser347Cys	P: PS2-very strong + PS4-moderate + PM2-supporting + PP2+PP3	Yes	Yes, PMIDs: 23754559, 27228464, 7874170, 11781872, 12884424, 20643727, 23348274, 25271085
P4	FGFR2, NM_000141.5, c.1026C>G, p.Cys342Trp	PS2-very strong + PS4-moderat + PM5+PM2-supporting + PP3-moderate	Yes	Yes, PMIDs: 16418739, 11173845, 11781872, 27803855, 8522336, 8528214, 12186468
P5	FGFR3, NM_000142.5, c.749C>G, p.Pro250Arg	PS2+PS4+PS3+PM2-supporting	Yes	Yes, PMIDs: 15241680, 8841188, 9042914
P6	TWIST1, NM_000474.4, c.415C>T, p.Pro139Ser	LP: PS4-moderate + PM2-supporting + PM5+PP3	From mother	Yes, PMID: 9585583
P7	ERF, NM_006494.4, c.787C>T, p.Gln263Ter	LP: PVS1-strong + PS2-moderate + PM2-supporting	Yes	NO
P8	del (11) (q23.3q25) (chr11:119627366–134,946,516)	P: 3C (0.9)+4 A (0.45)	Na	Yes, PMID: 8880580
P9	SON, NM_138,927.4, c.5753_c.5756del, p.Val1918 GlufsTer87	P: PVS1+PS2-very strong + PS4+PM2-supporting	Yes	Yes, PMIDs: 27545680, 27545676, 27256762
P10	HUWE1,NM_031407.7, c.12067C>T, p.Arg4023Cys	LP: PS4+PM2-supporting + PP3	From mother	Yes, PMIDs: 29180823, 36353900
P11	NOG, NM_005450.6, c.562G>T, p.Glu188Ter	LP: PVS1-strong + PS2-supporting + PM2-supporting	Yes	NO
P12	SRCAP, NM_006662.3, c.7330C>T, p.Arg2444Ter	P: PVS1-strong + PS2-very strong + PS4+PM2-supporting	Yes	Yes, PMIDs: 25433523, 23621943, 22265015, 22965468
P13	KRAS, NM_004985.5, c.40G>A, p.Val14Ile	P: PS2+PS3-moderate + PS4+PM1+PM2-supporting + PP2+PP3	From mother	Yes, PMIDs: 16474405, 17704260, 18958496, 19020799, 20949621
P14	EFNB1, NM_004429.5, c.708dup, p.Lys237 GlnfsTer82 FZD4, NM_012193.4, c.1511G>A, p.Trp504Ter	LP: PVS1-strong + PS2-moderate + PM2-supporting LP: PVS1-moderate + PS2+PM2-supporting + PP4	Yes; Yes	NO; NO
P15	FGFR3, NM_000142.5, c.1172C>A, p.Ala391GLu	P: PS2-very strong + PS3-supporting + PS4-moderate + PM2-supporting + PP3	From mother	Yes, PMIDs: 7493034, 8880573, 11426459, 20199409
P16	TWIST1, NM_000474.4, c.396_416dup, p.Lys133pro139dup	P: PS4+PM2-supporting + PM4+P P1-strong	From father	Yes, PMID: 20643727
P17	PQBP1, NM_001,032,382.2, c.459_462del, p.Arg153 SerfsTer41	P: PVS1+PS4+PM2-supporting	From mother	Yes, PMIDs: 20950397, 30500859, 31316545
P18	PTEN, NM_000314.8, c.72C>G, p.Asp24GLu	LP:PS4-supporting + PM2-supporting + PM5 +PP2+PP3	From mother	NO

No., number; Na, not available; P, pathogenic; LP, likely pathogenic.

Additionally, *KRAS*, *HUWE1*, *SON*, and an 11q23.3q25 microdeletion (previously reported to be associated with craniosynostosis) were identified in patients P9, P10, P13, and P8, respectively, with detailed clinical and genetic data provided in Tables 1, 3.

Three pathogenic variants in *PTEN*, *PQBP1*, and *SRCAP* were detected in patients P12, P17, and P18, respectively. All three probands presented with craniosynostosis along with other clinical manifestations; while megalocephaly, microcephaly, and skeletal anomalies are well-documented features of syndromes related to these genes, craniosynostosis represents a novel phenotypic feature.

**FIGURE 3:**
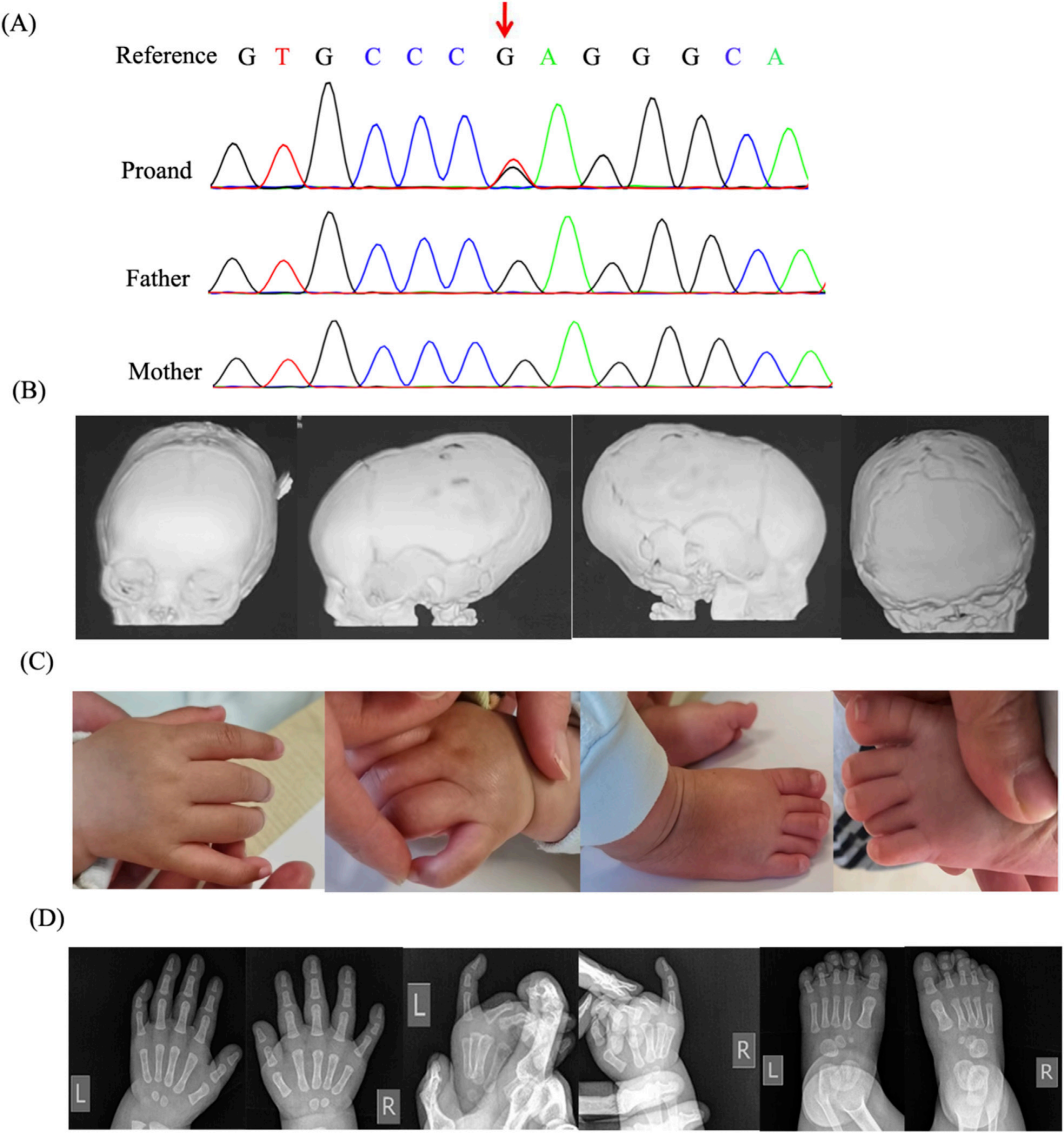
Clinical and genetic features of the proband (P11) harboring the *NOG* variant (c.562G>T, p. Glu188Ter). **(A)** Sanger sequencing confirming the *de novo* variant. **(B)** Head CT scan (3D reconstruction, bone window) showing scaphocephaly (elongated anterior-posterior axis), bilateral depression of the cranial vault, and premature fusion of the metopic suture, superior sagittal suture, and right coronal suture. **(C)** Clinical photograph of the hands demonstrating restricted flexion at the distal interphalangeal joints of the fifth fingers and absence of flexion creases. Clinical photograph of the feet showing upward deviation of the middle toes and absence of creases on toes 2–5. **(D)** Hand and foot radiographs showing no evidence of bony fusion or synostosis.

Furthermore, a heterozygous *de novo* nonsense variant (c.562G>T, p. Glu188Ter) in *NOG* (NM_005450.6) was identified in patient P11 (Figure 3A), who presented with scaphocephaly, craniosynostosis, skeletal abnormalities, and hypospadias (Figures 3B–D). Pathogenic *NOG* variants are typically associated with syndromes characterized by limb synostoses and hearing loss, with no previous reports linking them to cranial suture synostosis, thus supporting *NOG* as a novel candidate gene for SCS.

Among the 17 variants, four were novel, 10 occurred *de novo*, six were maternally inherited, and 1 was paternally inherited. Maternal carriers (patients P6, P10, P13, P15, P17, and P18) exhibited variable phenotypes, with some being clinically asymptomatic. In contrast, patient P16, who harbored a paternally inherited *TWIST1* variant, and his elder twin sister presented with identical symptoms, while their father only showed mild hypertelorism—findings indicative of high clinical heterogeneity even among individuals carrying the same variant.

### Functional analysis of key modules

**FIGURE 4:**
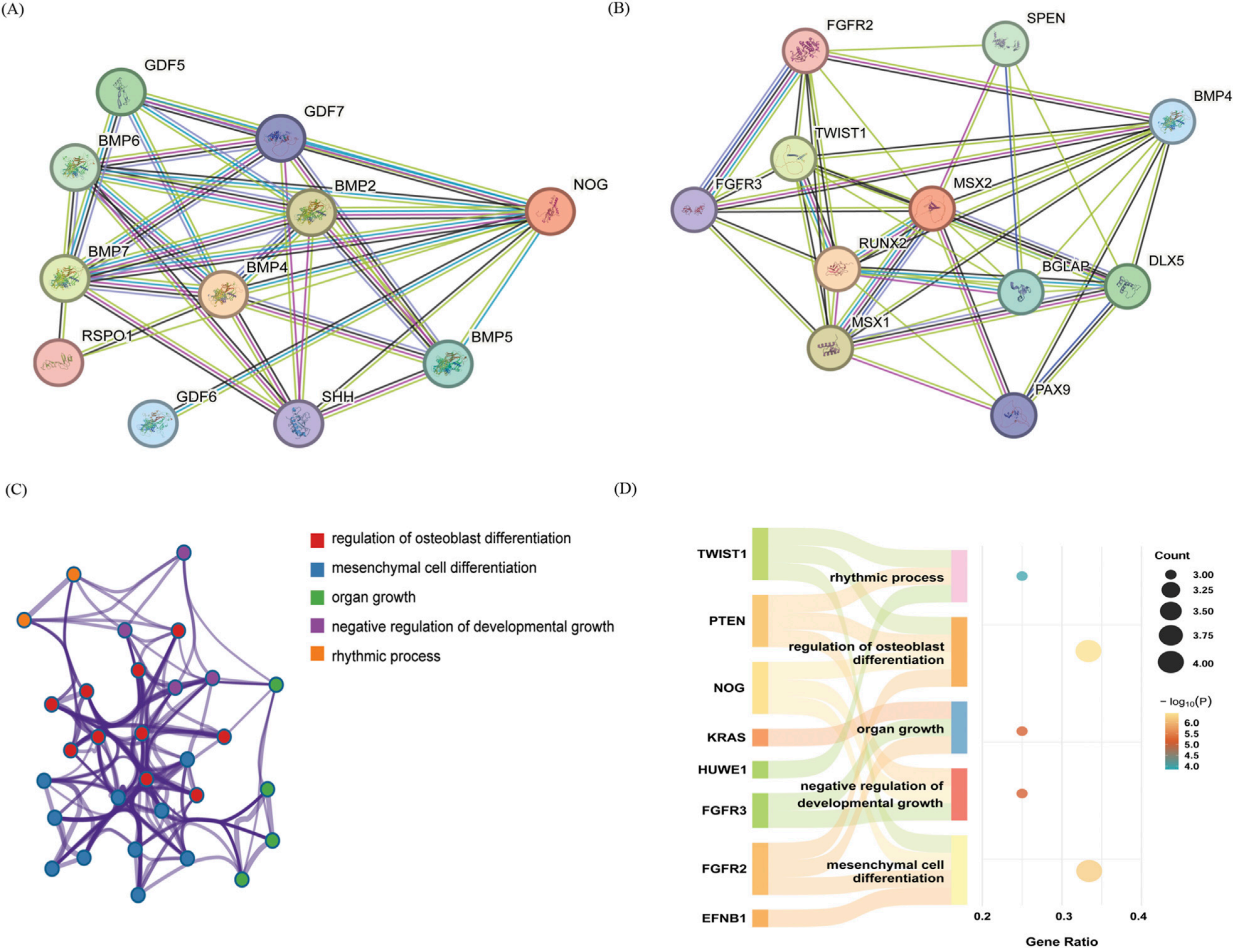
Gene regulatory network, functional enrichment network, and integrated Sankey-bubble analysis of key craniosynostosis genes. **(A,B)** Gene regulatory network of craniosynostosis-associated factors, through BMP4, NOG is linked to a set of well-characterized genes implicated in craniosynostosis pathogenesis (colored nodes: query proteins/first-shell interactors; white nodes: second-shell interactors; edges = protein-protein associations, cyan and magenta edges: known interactions; green, red, and blue edges: predicted interactions; light green, black and light blue edges: textmining, co-expression, and protein homology). **(C)** Metascape-generated enrichment network of significantly enriched GO biological processes (nodes = terms; edges = shared genes; colors = functional clusters). **(D)** Integrated Sankey diagram and bubble plot showing relationships between candidate genes, enriched biological processes, gene counts, and enrichment significance (bubble size = gene count; color intensity = significance), highlighting core pathways in abnormal osteoblast/mesenchymal differentiation and suture development in which NOG involved.

To explore the functional interaction of key genes identified in our study, we performed PPI network analysis using STRING database. As illustrated in Figures 4A,B, *NOG* was clustered with *BMP4*, which was further connected to well-established driver genes, including *FGFR2*, *FGFR3*, *TWIST1*, *RUNX2*, and *MSX2*.

Subsequently, to elucidate the biological functions underlying these PPI-derived core genes, we conducted functional enrichment analysis of all 12 core genes using the Metascape database. This analysis identified several significantly enriched and highly interconnected GO biological processes (Figures 4C,D; Table 4), which were closely associated with skull development, osteogenic differentiation, and tissue growth regulation—core biological events governing cranial suture patency and fusion.

**TABLE 4:** Enriched Gene Ontology biological process terms for the 12 genes.

Category	Term	Count	%	List total	P Value	Genes	Fold enrichment
GOTERM_BP_DIRECT	Mesenchymal cell differentiation	4	33.33	12	5.54E-07	EFNB1, FGFR2, TWIST1, NOG	148.1863
GOTERM_BP_DIRECT	Negative regulation of developmental growth	3	25	12	6.69E-06	FGFR3, PTEN, NOG	78.72396
GOTERM_BP_DIRECT	Organ growth	3	25	12	6.28E-06	FGFR3, FGFR2, KRAS	80.39894
GOTERM_BP_DIRECT	Regulation of osteoblast differentiation	4	33.33	12	3.62E-07	FGFR2, PTEN, TWIST1, NOG	137.4091
GOTERM_BP_DIRECT	Rhythmic process	3	25	12	0.000141	PTEN, TWIST1, HUWE1	28.30524

An integrated Sankey diagram combined with a bubble plot (Figure 4D; Table 4) further systematically depicted the correspondence between key hub genes and the enriched biological processes. The enriched gene set mainly included *NOG*, *FGFR2*, *FGFR3*, *TWIST1*, *PTEN*, *KRAS*, *EFNB1*, and *HUWE1*, among which *FGFR2*, *FGFR3*, and *TWIST1* are well-characterized causative genes of craniosynostosis. Notably, *NOG* was significantly enriched in pathways regulating both osteoblast differentiation and mesenchymal cell differentiation, which strongly supports its functional relevance to cranial suture development.

## Discussion

Craniosynostosis is characterized by substantial genetic heterogeneity, which complicates molecular diagnosis due to incomplete penetrance, variable expressivity, and weak genotype-phenotype correlations. The core genetic finding of this study is the identification of pathogenic/likely pathogenic variants in 12 genes (*FGFR2*, *FGFR3*, *TWIST1*, *EFNB1*, *ERF*, *SON*, *HUWE1*, *KRAS*, *PTEN*, *PQBP1*, *SRCAP*, and *NOG*) and one 11q23.3q25 microdeletion in 18 SCS patients. Notably, *FGFR2* contributed the highest diagnostic yield (22.22%), followed by *FGFR3* and *TWIST1* (13.33% each), while the remaining nine genes were each associated with a single patient. Most importantly, our study is the first to report an association between *PTEN*, *PQBP1*, *SRCAP*, and *NOG* and craniosynostosis, which significantly expands the genetic spectrum of this disorder.

Clinically, craniofacial dysmorphism was the most prevalent symptom (100%), followed by neurodevelopmental impairment (72.22%) and ocular defects (61.11%). We observed significant expansion of systemic involvement, including congenital heart defects (27.78%, primarily atrial septal defect/patent ductus arteriosus), genitourinary defects (27.78%, e.g., cryptorchidism/hypospadias), and digital/acral anomalies (22.22%). These findings support the hypothesis of multi-germ layer (ectodermal/mesodermal) disruption in SCS pathogenesis. Additionally, trans-system phenotypic associations (e.g., eye-craniofacial, neuro-cardiovascular) further corroborate multi-germ layer involvement. Practically, these observations highlight the need for universal neurodevelopmental and ophthalmological assessments, cardiovascular screening for SCS patients, prioritization of genetic testing combined with neuroimaging for precision diagnosis and management, and leveraging specific phenotypic combinations (e.g., SCS + hypospadias + digital/acral anomalies) to facilitate novel gene discovery.

Consistent with established literature (Yapijakis et al., 2023; Timberlake et al., 2023), disruptions in FGF-FGFR, TGF-β/BMP, Wnt, and ephrin signaling—mediated by variants in *FGFR1-3*, *TWIST1*, *MSX2*, *EFNB1*, *ERF*, *RUNX2*, and *TCF12*—are major causes of craniosynostosis (Wu and Gu, 2019; Yapijakis et al., 2023). In our cohort, variants in *FGFR2*, *FGFR3*, *TWIST1*, *ERF*, and *EFNB1* accounted for over half of genetically resolved cases, reinforcing the predominance of FGF/TWIST and BMP/MSX2 pathway dysregulation in SCS (Yapijakis et al., 2023). These genes are linked to well-defined syndromes: *FGFR1-3* variants cause Pfeiffer, Crouzon, Apert, and Muenke syndromes; *TWIST1* variants underlie Saethre-Chotzen syndrome and Craniosynostosis type 1; *ERF* variants (encoding an ETS2 repressor factor in FGF/TWIST pathways (Yapijakis et al., 2023; Armand et al., 2019), typically cause Craniosynostosis 4 (OMIM#600775). ERF truncating variants impair nuclear-cytoplasmic shuttling (via disrupted ERK interaction/C-terminal domains) and abrogate osteoblast proliferation repression, a mechanism likely underlying craniofacial abnormalities (Goto et al., 2025). Our novel *ERF* nonsense variant (P7, p. Gln263Ter) is predicted to exert a similar loss-of-function effect.

Trio sequencing yielded a critical finding: a high *de novo* variant rate (10/18, 55.56%), which underscores its high clinical utility for SCS molecular diagnosis. Additionally, we observed maternal inheritance in 27.78% of cases (*FGFR3*, *KRAS*, *TWIST1*, *HUWE1*, *PTEN*, *PQBP1*), highlighting notable inheritance risks—particularly in cases with mild maternal phenotypes (e.g., midface hyperplasia in P15’s mother, valvulopathy in P13’s mother, slightly enlarged head circumference in P17’s mother). This finding emphasizes the importance of family-based genetic analysis to inform accurate genetic counseling and risk assessment for SCS families.

Four probands (P13, P9, P10, P8) harbored pathogenic variants/CNV associated with known craniosynostosis-related syndromes: *KRAS* (Noonan syndrome 3, OMIM #609942), *SON* (ZTTK syndrome, OMIM 617140), *HUWE1* (Turner type, OMIM 309590), and 11q23.3q25 microdeletion (Jacobsen syndrome, OMIM 147791). *KRAS* (RAS/MAPK pathway) may disrupt skull development via constitutive activation when mutated (Ueda et al., 2017). *SON* (RNA splicing cofactor) and *HUWE1* are craniosynostosis risk genes, highly expressed in cranial neural crest cells and converging on regulatory networks controlling osteoblast differentiation (Timberlake et al., 2023; Moortgat et al., 2018). While craniosynostosis is a known feature of Jacobsen syndrome (Linares et al., 2016), the mechanism linking 11q23.3q25 microdeletion to suture fusion remains unclear and requires further investigation.

One of the most important novel findings of this study is the identification of craniosynostosis in patients harboring variants in *PTEN* (P18), *PQBP1* (P17), and *SRCAP* (P12)—a phenotype never previously reported for these genes. For *PTEN*, functional enrichment analysis (Figure 4D) confirms its involvement in osteoblast differentiation regulation and developmental growth suppression, and we propose it may contribute to craniosynostosis by inhibiting PI3K/AKT signaling to regulate neural crest development and craniofacial morphogenesis (Yang et al., 2018). For *PQBP1*, its knockdown in developing mesoderm (bone/cartilage precursor) inhibits FGF-induced target gene expression and *Fgf4* levels (Yang et al., 2020), suggesting it may contribute to SCS pathogenesis via regulating FGF/FGFR signaling—a hypothesis requiring further validation. For *SRCAP*, its role in histone H2A.Z deposition (Johal et al., 2025) implicates epigenetic dysregulation in suture biology, potentially converging with mechanisms involving other chromatin modifiers (e.g., *HUWE1*) (Beacon et al., 2021).

The most striking finding of our study is the identification of a *de novo NOG* nonsense variant (p.Glu188Ter) in SCS patient P11. *NOG* encodes noggin, a potent BMP antagonist critical for cranial suture patency and skull vault development, and dysregulated BMP signaling is a well-recognized cause of craniosynostosis (Warren et al., 2003; Maxson and Ishii, 2008; Whitton et al., 2016; Komatsu et al., 2013). Reduced noggin expression is consistently documented in fused human sutures and craniosynostosis models, while noggin application prevents pathological fusion in animals (Wu and Gu, 2019; Cooper et al., 2007; Shen et al., 2009; Gabbay et al., 2006; Hel et al., 2007; Al-Rekabi et al., 2017; Jacob et al., 2007; Sanchez-Lara et al., 2010; Wang et al., 2019). Studies on Nog^−/−^ lacZ mice have further estabolished noggin as a BMP antagonist that governs dorsoventral patterning of the neural tube, somite differentiation, as well as limb cartilage and joint formation, with loss of Noggin resulting in multiple axial and appendicular skeletal malformations (McMahon et al., 1998; Brunet et al., 1998). Moreover, Noggin had been shown to regulate cranial suture fusion in postnatal mice (Warren et al., 2003). Cnosistently, the Mouse Genome Informatics (MGI) database (https://www.informatics.jax.org) documents that Nog mutant mice exhibit craniosynostosis accompanied a spectrum of craniofacial defects—including frontal bossing, brachyturricephaly, dolichocephaly, wide anterior fontanel, micrognathia, maxillary hypoplasia, shallow orbits, microcephaly, prominent forehead, and palatal abnormalities—as well as skeletal involvement across the basicranium, vertebrae, cartilage, joints, and bone mineralization. Collectively, these MGI-derived phenotypes provide *in vivo* evidence reinforcing the essential role of *NOG* in cranial suture patency and skeletal development, further supporting the pathogenicity of the p. Glu188Ter variant identified in our patient. Our functional analyses provide direct support for this novel association: PPI network analysis showed *NOG* directly interacts with *BMP4*, which connects to established craniosynostosis-related genes (*FGFR1-3*, *MSX2*, *RUNX2*, *TWIST1*), implicating *NOG* in key pathogenic signaling pathways; functional enrichment analysis confirmed *NOG* is significantly enriched in osteoblast and mesenchymal cell differentiation regulation. This is the first human genetic evidence linking *NOG* dysfunction to craniosynostosis, expanding its phenotypic spectrum and identifying dysregulated BMP antagonism as a novel pathogenic mechanism. Further functional studies are needed to elucidate *NOG*’s precise role in suture biology.

## Conclusion

In conclusion, our genetic analysis identified causative variants and one CNV in 18 Chinese craniosynostosis probands, including four novel variants and novel gene associations. Acknowledging limitations, retrospective design and small sample size, our findings reinforce craniosynostosis’ genetic heterogeneity, including established pathways (e.g., FGF/TWIST) and diverse syndromic genes. Critically, we implicate *PTEN*, *PQBP1*, and *SRCAP* in a novel craniosynostosis phenotype and identify *NOG* as a new candidate gene for craniosynostosis. These findings expand the genotypic and phenotypic spectrum of craniosynostosis and provide new avenues for pathogenesis research.

## Data Availability

The raw data supporting the conclusions of this article will be made available by the authors, without undue reservation.
